# DeWitt County Medical Society

**Published:** 1874-05-01

**Authors:** C. Goodbrake

**Affiliations:** Secretary


DEWITT COUNTY MEDICAL SOCIETY.—This Society met in annual session, at the office of Dr. W. W. Adams, in Clinton, on Tuesday, the 14th day of April, 1874. There was a good attendance, and the proceedings were very interesting.

The election of officers being in order, the following gentlemen were elected for the ensuing year:

*President*, T. K. Edmiston, M.D.; *Vice-President*, J. J. Lake, M.D.; *Secretary*, C. Goodbrake, M.D.; *Treasurer*, John Wright, M.D.; *Censors*, Drs. W. W. Adams, W. G. Cochran, and Z. H. Madden.

*Delegates to the Illinois State Medical Society*—Drs. John Wright, J. S. Miller, and F. H. Tatman.

*Delegates to the American Medical Association*—Drs. J. H. Tyler, and J. J. Lake.

C. GOODBRAKE, M.D.,
*Secretary.*

